# Clinical indications for image-guided interventional procedures in the musculoskeletal system: a Delphi-based consensus paper from the European Society of Musculoskeletal Radiology (ESSR)—part V, knee

**DOI:** 10.1007/s00330-021-08258-1

**Published:** 2021-09-14

**Authors:** Luca Maria Sconfienza, Miraude Adriaensen, Domenico Albano, Andrea Alcala-Galiano, Georgina Allen, Maria Pilar Aparisi Gómez, Giacomo Aringhieri, Alberto Bazzocchi, Ian Beggs, Vito Chianca, Angelo Corazza, Danoob Dalili, Miriam De Dea, Jose Luis del Cura, Francesco Di Pietto, Elena Drakonaki, Fernando Facal de Castro, Dimitrios Filippiadis, Salvatore Gitto, Andrew J. Grainger, Simon Greenwood, Harun Gupta, Amanda Isaac, Slavcho Ivanoski, Monica Khanna, Andrea Klauser, Ramy Mansour, Silvia Martin, Vasco Mascarenhas, Giovanni Mauri, Catherine McCarthy, David McKean, Eugene McNally, Kalliopi Melaki, Rebeca Mirón Mombiela, Ricardo Moutinho, Marina Obradov, Cyprian Olchowy, Davide Orlandi, Raquel Prada González, Mahesh Prakash, Magdalena Posadzy, Saulius Rutkauskas, Žiga Snoj, Alberto Stefano Tagliafico, Alexander Talaska, Xavier Tomas, Violeta Vasilevska-Nikodinovska, Jelena Vucetic, David Wilson, Federico Zaottini, Marcello Zappia, Carmelo Messina

**Affiliations:** 1 IRCCS Istituto Ortopedico Galeazzi, Via Riccardo Galeazzi 4, 20161 Milan, Italy; 2 Dipartimento Di Scienze Biomediche Per La Salute, Università Degli Studi Di Milano, Milan, Italy; 3 Department of Medical Imaging, Zuyderland Medical Center, Sittard-Geleen, Heerlen, Brunssum, Kerkrade the Netherlands; 4 Sezione Di Scienze Radiologiche, Dipartimento Di Biomedicina, Neuroscienze E Diagnostica Avanzata, Università Degli Studi Di Palermo, Palermo, Italy; 5 Hospital Universitario, 12 de Octubre, Madrid, Spain; 6 St Luke’s Radiology Oxford Ltd, Oxford, UK; 7 University of Oxford, Oxford, UK; 8 Department of Radiology, Auckland City Hospital, Auckland, New Zealand; 9 Department of Radiology, Hospital Vithas Nueve de Octubre, Valencia, Spain; 10 Diagnostic and Interventional Radiology, Department of Translational Research and New Technologies in Medicine and Surgery, University of Pisa, Pisa, Italy; 11 Diagnostic and Interventional Radiology, IRCCS Istituto Ortopedico Rizzoli, Bologna, Italy; 12 Analytic Imaging, Edinburgh, UK; 13 Ospedale Evangelico Betania, Napoli, Italy; 14 Clinica Di Radiologia EOC IIMSI, Lugano, Switzerland; 15 School of Biomedical Engineering & Imaging Sciences, King’s College London, London, UK; 16 Studio MSK, Belluno, Italy; 17 Hospital Universitario Donostia, San Sebastian, Spain; 18 Dipartimento Di Diagnostica Per Immagini, Pineta Grande Hospital, Castel Volturno, Italy; 19 MSK Radiology Services, Heraklion, Crete Greece; 20 Hospital General Universitario de Valencia, Valencia, Spain; 21 2nd Department of Radiology, University General Hospital “ATTIKON” Medical School, National and Kapodistrian University of Athens, Haidari, Athens, Greece; 22 Cambridge University Hospitals, Cambridge, UK; 23 Mid Cheshire Hospitals NHS Foundation Trust, Cheshire, UK; 24 Leeds Teaching Hospitals, Leeds, UK; 25 Guy’s and St Thomas’ Hospitals, London, UK; 26 Department of Radiology, Special Hospital for Orthopedic Surgery and Traumatology, St. Erazmo, Ohrid, North Macedonia; 27 Medical Faculty, University Ss. Cyril and Methodius in Skopje, Skopje, Macedonia; 28 Imperial College Healthcare NHS Trust, London, UK; 29 Department of Radiology, Medical University of Innsbruck, Innsbruck, Austria; 30 Oxford Musculoskeletal Radiology, Oxford University Hospitals, Oxford, UK; 31 Hospital Universitario Son Llatzer, Palma, Spain; 32 Musculoskeletal Imaging Unit, Hospital da Luz, Lisbon, Portugal; 33 AIRC, Advanced Imaging Research Consortium, Lisbon, Portugal; 34 Division of Interventional Radiology, Istituto Europeo Di Oncologia IRCCS, Milan, Italy; 35 Department of Oncology and Hemato-Oncology, University of Milano, Milan, Italy; 36 Oxford Musculoskeletal Radiology, Oxford, UK; 37 Buckinghamshire Healthcare NHS Trust, Aylesbury, UK; 38 Department of Radiology, Charité-Universitätsmedizin Berlin, Berlin, Germany; 39 Department of Radiology, Herlev og Gentofte Hospital, Herlev, Denmark; 40 Hospital de Loulé, Loulé, Portugal; 41 Present Address: Department of Radiology, Sint Maartenskliniek, Nijmegen, The Netherlands; 42 Department of Oral Surgery, Wroclaw Medical University, Wroclaw, Poland; 43 Department of Radiology, Ospedale Evangelico Internazionale, Genoa, Italy; 44 Hospital POVISA, Vigo, Spain; 45 Post Graduate Institute of Medical Education & Research (PGIMER), Chandigarh, India; 46 Indywidualna Praktyka Lekarska Magdalena Posadzy, Poznan, Poland; 47 Department of Radiology, Lithuanian University of Health Sciences, Kaunas, Lithuania; 48 Institute of Radiology, University Medical Centre Ljubljana, Zaloska 7, 1000 Ljubljana, Slovenia; 49 Faculty of Medicine, University of Ljubljana, Ljubljana, Slovenia; 50 Department of Health Sciences, University of Genova, Genoa, Italy; 51 IRCCS Ospedale Policlinico San Martino, Genoa, Italy; 52 Department of Radiology, AUVA Trauma Center, Vienna, Austria; 53 Radiology Dpt. MSK Unit. Hospital Clinic (CDIC), University of Barcelona (UB), Barcelona, Spain; 54 University Institute of Radiology in Skopje, Clinical Center “Mother Theresa”, Skopje, Macedonia; 55 Radiology Department, Hospital ICOT Ciudad de Telde, Las Palmas, Spain; 56 Department of Medicine and Health Sciences, University of Molise, Campobasso, Italy; 57 Varelli Institute, Naples, Italy

**Keywords:** Interventional radiology, Knee, Patellar tendon, Platelet-rich plasma, Hyaluronic acid

## Abstract

**Objectives:**

Interventional procedures around the knee are widely adopted for treating different musculoskeletal conditions. A panel of experts from the Ultrasound and Interventional Subcommittees of the European Society of Musculoskeletal Radiology (ESSR) reviewed the existing literature to assess the evidence on image-guided musculoskeletal interventional procedures around the knee, with the goal of highlighting some controversies associated with these procedures, specifically the role of imaging guidance, as well as the efficacy of the medications routinely injected.

**Methods:**

We report the results of a Delphi-based consensus of 53 experts in musculoskeletal radiology, who reviewed the published literature for evidence on image-guided interventional procedures around the knee to derive a list of pertinent clinical indications.

**Results:**

A list of 10 statements about clinical indications of image-guided procedures around the knee was created by a Delphi-based consensus. Only two of them had the highest level of evidence; all of them received 100% consensus.

**Conclusions:**

Ultrasonography guidance is strongly recommended for intra-articular and patellar tendinopathy procedures to ensure the precision and efficacy of these treatments. Prospective randomized studies remain warranted to better understand the role of imaging guidance and assess some of the medications used for interventional procedures around the knee.

**Key Points:**

*• A list of 10 evidence-based statements on clinical indications of image-guided interventional procedures around the knee was produced by an expert panel of the ESSR.*

*• Strong consensus with 100% agreement was obtained for all statements.*

*• Two statements reached the highest level of evidence, allowing us to strongly recommend the use of ultrasonography to guide intra-articular and patellar tendon procedures to ensure higher accuracy and efficacy of these treatments.*

**Supplementary Information:**

The online version contains supplementary material available at 10.1007/s00330-021-08258-1.

## Introduction

Interventional procedures around the knee are widely utilized to treat different musculoskeletal conditions. Some of these interventions (e.g., injections or aspirations) are often performed without image guidance, particularly by orthopedists. However, image guidance ensures correct needle position for optimal medication delivery, avoiding injuries to adjacent neurovascular bundles [1–5]. Currently, image guidance for musculoskeletal procedures around the knee has not been incorporated into established guidelines, due to sparse and contentious evidence on its clinical impact in the literature. Moreover, the choice of the procedure is debatable, as new approaches, such as the use of hyaluronic acid (HA) and platelet-rich plasma (PRP), have been recently introduced to treat both joint and tendon conditions [6–8]. Accordingly, the Ultrasound (US) and Interventional Subcommittees of the European Society of Musculoskeletal Radiology (ESSR), together with its Research Committee, initiated in 2019 a collaborative task dedicated to reviewing the existing literature on image-guided musculoskeletal interventional procedures in the lower limb and providing evidence for its clinical indications. This paper reports the list of statements provided by an expert panel of the ESSR and obtained by a Delphi process on published literature evaluating image-guided interventions around the knee.

## Materials and methods

Institutional review board approval was not needed as no patient-specific data were involved. This paper concludes the task carried on by an expert panel of the ESSR which reviewed the evidence of image-guided musculoskeletal interventional procedures in the lower limb. Here, we report the results focusing on tendon, joint, and bursal interventions around the knee. As previously done [9–12], we used a literature-based Delphi process of evidence review including multiple discussion rounds to evaluate the opinion of experts on debatable topics, drafted on the basis of the existing literature, to obtain a final shared agreement [13]. The AGREE II tool was followed to guarantee the quality of this analysis [14]. Supplementary material includes the explanation of the Delphi method steps. The Oxford Centre for Evidence-based Medicine evidence levels were employed to assess the evidence of published papers [15].

## Results

1. **Intra-articular US-guided procedures around the knee joint, such as arthrocentesis and intra-articular injections, are more accurate than palpation-guided procedures, resulting in improved fluid aspiration and injection therapeutic outcome(s)**.

Level of evidence, 1

Agree, *n* = 53; disagree, *n* = 0; abstain, *n* = 0. Agreement = 100%

A systematic review reported that image-guided and particularly US-guided knee injections are more accurate than palpation-guided procedures [16]. A randomized trial also reported superior accuracy of the US-guided technique performed by trainees as compared with palpation-guided procedures performed by experienced clinicians [17]. Accuracy rates were high (95–100%) and similar when injecting the joint through in-plane superolateral, mid-lateral, and mid-medial approaches [18]. Accuracy rates were also high (95%) using an out-of-plane technique with mid-lateral [19] and mid-medial [20] approaches—although some authors reported that an out-of-plane mid-medial approach could result in decreased accuracy [19]. Similarly, accuracy rates of US-guided knee arthrocentesis have been reported to be superior to palpation guidance [21]. Most studies reported superior injection benefits [22] and + 183% improved joint aspiration with improved 2-week outcome [23] using US-guided rather than palpation-guided procedures. Randomized trials comparing US-guided and palpation-guided corticosteroid intra-articular injections [17, 22] reported pain reduction at 2-week follow-up, 107% increase in the responder rate, and 36% increase in therapeutic duration with subsequent cost reduction, using US guidance [22]. In knee osteoarthritis treated with HA injections, the use of US guidance resulted in enhanced functional and pain-score improvement after 6 and 12 weeks [24], with long-term decreased knee arthroplasty rate [25] when compared to palpation guidance. In emergency settings, both blind and US-guided arthrocentesis were successful, although the latter led to higher volume aspiration for novice practitioners [26].

2. **US-guided knee joint injections of corticosteroid-anesthetic give short-to-midterm pain relief and functional improvement in inflammatory arthritis. Although similar outcomes may be observed in osteoarthritis, efficacy is controversial, and alternative analgesic therapies (such as oxygen-ozone) have been proposed, but evidence supporting their use remains limited.**

Level of evidence, 2

Agree, *n* = 53; disagree, *n* = 0; abstain, *n* = 0. Agreement = 100%

In a randomized study, US-guided injections of corticosteroid-anesthetic demonstrated pain relief and improved function at 2 and 6 weeks in patients with inflammatory arthritis [17]. In knee osteoarthritis, US-guided intra-articular administration of corticosteroid-anesthetic has been shown to produce similar clinical and functional outcomes at 1, 2, and 4 weeks in a randomized study compared to oxygen-ozone injection [27], as well as in a cohort study [28]. However, no outcome difference was found in another randomized trial comparing US-guided injections of placebo or corticosteroid at 2-week follow-up [29]. These findings are consistent with a systematic review and meta-analysis including both US-guided and palpation-guided injections, where no clear clinical advantage of corticosteroids use was found in the short-to-midterm [30]. Furthermore, repeated corticosteroid use has been reported to accelerate cartilage volume loss in a 2-year clinical trial that randomized patients with knee osteoarthritis to triamcinolone or placebo injections [31]. Hence, current evidence supports the use of US-guided injections of corticosteroid-anesthetic in inflammatory arthritis, but it is contradictory about their efficacy and cautious about the long-term safety of repeated injections in knee osteoarthritis.

Regarding other US-guided intra-articular analgesic treatments, a randomized controlled study compared oxygen-ozone efficacy with that of corticosteroid at 1 week, 1 month, and 3 months [27]. Both therapies were effective in improving symptoms and functional outcomes at 1 week and 1 month. This improvement was sustained for 3 months in patients treated with oxygen-ozone but not with corticosteroids [27].

3. **US-guided HA intra-articular injections are safe and improve pain scores and function in knee osteoarthritis, showing greater efficacy than steroids in the long term.**

Level of evidence, 3

Agree, *n* = 53; disagree, *n* = 0; abstain, *n* = 0. Agreement = 100%

Systematic reviews of overlapping meta-analyses found that HA injections are effective in treating knee osteoarthritis with no increased risk of adverse events [32], with positive effects lasting up to 26 weeks [33]. Most studies used blind techniques to perform injections. Nevertheless, a case–control retrospective study reported that US-guided HA injection improved pain and function at 6-month follow-up [34]. Greater pain reduction was observed compared to intra-articular corticosteroid injections [34]. These findings are concordant with a previous meta-analysis including high-quality randomized trials using intra-articular corticosteroid and HA injections, regardless of the injection approach [35]. Efficacy on pain was greater in the corticosteroid group in the short term (up to 1 month), similar in midterm (3 months), and greater in the HA group in the long term (6 months) [35]. US guidance improved injection accuracy and clinical outcomes at 6- and 12-week follow-up compared with palpation-guided injections in a randomized trial [24]. Long-term, precise intra-articular injection of HA by US guidance was associated with a reduced knee arthroplasty rate compared to the palpation-guided approach, particularly in obese patients [25, 32].

4. **US-guided injections of regenerative medications have been reported to show clinical benefit by relieving pain and enhancing function in patients with knee osteoarthritis but lack randomized controlled trial evidence.**

Level of evidence, 3

Agree, *n* = 53; disagree, *n* = 0; abstain, *n* = 0. Agreement = 100%

Regenerative therapies have emerged as alternative strategies to treat knee osteoarthritis. Among them, blood derivatives such as PRP [36], autologous conditioned serum [37], and autologous protein solution [38] intra-articular administration under US guidance safely produced clinical improvement observed early after treatment and sustained up to 1 year thereafter [36–38]. Adding growth hormone to PRP improved joint function in the short term [39]. However, the efficacy of blood derivatives remains controversial. A randomized controlled trial reported similar improvements up to 6 months after saline injection [38]. Larger randomized controlled studies providing longer-term follow-up are needed.

US-guided injections of other regenerative substances, such as adipose-derived stem cells [40] and amniotic membrane/umbilical cord particulate [41], have shown clinical and functional improvements up to 6 and 12 months after treatment in non-controlled studies on small series. Evidence regarding their clinical use remains limited. Prolotherapy has been reported to be less effective than PRP in reducing pain and functional limitation in patients with knee osteoarthritis [36].

5. **US-guided procedures around the menisci are promising for short-term pain management, but evidence supporting their use is limited**.

Level of evidence, 4

Agree, *n* = 53; disagree, *n* = 0; abstain, *n* = 0. Agreement = 100%

In patients with knee osteoarthritis and meniscal extrusion [42], tear, or degeneration [43], US-guided meniscus-targeted corticosteroid injections have been reported to relieve pain at short-term follow-up (at 1–4 weeks [42] and 5–6 weeks on average [43]). The absence of a control group is the main limitation of these studies. However, these preliminary findings encourage future higher-quality research. US-guided drainage of meniscal cysts with subsequent corticosteroid and anesthetic injection has been proposed as a safe and well-tolerated option to delay surgery, with complete symptom resolution reported at an average follow-up of 10 months in more than half of patients [44]. Higher-quality studies with longer-term follow-up are absent.

6. **In fat pad–related anterior knee pain syndromes, US-guided corticosteroid-anesthetic injection and fat pad alcohol ablation might be safe and effective in short-term pain reduction, although no randomized studies are available.**

Level of evidence, 4

Agree, *n* = 53; disagree, *n* = 0; abstain, *n* = 0. Agreement = 100%

Anterior knee pain may be associated with inflammation, hypertrophy, or edema of fat pads resulting in impingement syndromes [45, 46]. US-guided corticosteroid-anesthetic injections are safe and effective in the short-term reduction of pain secondary to suprapatellar fad pad inflammation [45]. A non-randomized study compared physical therapy with and without prior US-guided corticosteroid-anesthetic injection for suprapatellar fat-pad edema, showing a greater pain reduction in the injected patients at 1-month follow-up but not at 6-month follow-up [45]. Furthermore, an uncontrolled study on 12 patients with infrapatellar fat pad impingement syndrome evaluated the efficacy of serial US-guided ethanol-bupivacaine injections, resulting in pain reduction at 6-week follow-up [46]. However, the evidence is still limited.

7. **US-guided dry needling is effective in improving function and pain in patellar tendinopathy (PT), especially if associated with PRP. Conflicting results about the clinical effectiveness of PRP in PT do not allow supporting the use of this treatment as a first-line approach.**

Level of evidence, 1

Agree, *n* = 53; disagree, *n* = 0; abstain, *n* = 0. Agreement = 100%

A meta-analysis on nonsurgical approaches for PT confirmed that dry needling was one of the non-invasive treatments demonstrating clinical improvement [47]. Retrospective and case studies showed clinical improvement at 4 weeks in around 74% of cases [48–51]. Housner reported excellent to good satisfaction scoring at 4 weeks in 81% of 47 patients with recalcitrant PT with, however, one tendon rupture [49]. Pain improvement for refractory PT was reported combining two dry needlings with autologous blood injections performed 4 weeks apart [52]. US-guided PRP injection combined with dry needling and eccentric loading exercise was shown to be more effective than dry needling alone in refractory PT at 12 weeks [53]. Furthermore, dry needling was more effective than eccentric loading exercises alone [53]. PRP injection was more effective than extracorporeal shockwave therapy at 6 and 12 months follow-up [54]. High-volume and PRP injections were reported as having similar efficacy in the short term, while positive effects of high-volume injections gradually diminished and PRP showed greater efficacy in the medium term. However, their combination provided better results at 6 months [55]. Conversely, one study showed PRP alone had a similar clinical effect at 12 weeks when compared to saline [56].

8. **Other US-guided treatments have been shown to be safe for treating PT (corticosteroid, high-volume injections, prolotherapy, sclerosing injections with polidocanol, and HA). However, no studies compared them; thus clinical superiority of one treatment over another still needs clarification.**

Level of evidence, 3

Agree, *n* = 53; disagree, *n* = 0; abstain, *n* = 0. Agreement = 100%

Peritendinous corticosteroid injection for PT has been shown to be more effective than placebo at 3 months. However, it caused reversible short-term skin atrophy in 37% [57]. Further, some patients presented symptoms of relapse within 6 months when combined with aggressive rehabilitation.

US-guided high-volume injection has shown good short-term results in athletes and nonathletes [58, 59]. US-guided high-volume corticosteroid-anesthetic and saline injection have shown good results in PT [58, 59]. One study recommended post-injection physical therapy using eccentric loading [59]. Conversely, ambiguous results have been reported using high-volume image-guided injection for PT, with all patients showing clinical improvement, but 6/28 patients required surgery after treatment and 2/28 had additional corticosteroid injection [60].

Hyperosmolar dextrose prolotherapy can be safely used to treat intractable Osgood-Schlatter’s disease and chronic PT in young adolescents and adults. Some evidence suggests greater symptom improvement after prolotherapy than the usual conservative treatment [61, 62]. However, a randomized controlled trial on 49 knees with Osgood-Schlatter’s disease failed to show any pain difference after prolotherapy compared to lidocaine injection alone [63].

Small-cohort randomized controlled studies on sclerosing injections on groups of athletes and nonathletes reported improvement in knee function with short-term pain reduction [64, 65]. Moreover, this technique seems to offer long-term pain relief [66, 67].

US-guided HA injection is a safe and feasible treatment for PT pain [68]. US-guided peritendinous injections of HA performed on three occasions 1 week apart were safe, showing pain relief, a decrease in tendon thickness, and decreasing neovascularization at 3 weeks [69]. In a small series, 3 weeks of HA peritendinous injections showed a reduction in swelling and tenderness without adverse events [70].

9. **US-guided aspiration, wall fenestration, and corticosteroid injection of Baker’s cysts are safe and effective procedures in relieving pain and reducing cyst volume in patients with Baker’s cysts secondary to internal knee derangement.**

Level of evidence, 3

Agree, *n* = 53; disagree, *n* = 0; abstain, *n* = 0. Agreement = 100%

Although Baker’s cyst therapy depends on the primary cause, percutaneous interventions can be safely performed under US guidance [71–73]. In patients with knee osteoarthritis, US-guided Baker’s cyst aspiration and corticosteroid-anesthetic injection have shown significant pain relief and cyst diameters decrease up to 4 [71, 72] and 8 weeks [73]. Direct Baker’s cyst injection gave a greater size reduction and better clinical outcomes compared to an anterior knee joint injection at 4- and 8-week follow-up [73]. Aspiration and corticosteroid injection performed better than stand-alone physical therapy, even though their combination further ameliorated symptoms [72]. At long-term follow-up in patients with knee osteoarthritis [74] or other knee pathologies [75], US-guided aspiration, wall fenestration in the case of multilocular cysts, and injection of anesthetic and corticosteroid showed similar clinical benefit by relieving pain [74, 75] and reducing cyst volume [74]. A significant correlation exists between volume reduction and clinical improvement [74]. Baker’s cyst recurrence was noted in complex cysts, without any significant pain change between the patients with simple and complex cysts [74]. Close follow-up may be advantageous so the treatment can be repeated in case of recurrence [74]. In two case reports, US-guided sclerotherapy with hypertonic dextrose has been used as a treatment option for Baker’s cyst treatment [76, 77], but the clinical value has not still been demonstrated. US-guided intervention in Baker’s cyst when visually observed by the patient can be used as a positive bio-feedback, favorably affecting the treatment outcome [78].

10. **US-guided corticosteroid injections are more effective than blind injections to treat pes anserinus bursitis, but the added value of imaging to guide other periarticular injections (excluding patellar tendon and Baker’s cyst) has not been demonstrated.**

Level of evidence, 3

Agree, *n* = 53; disagree, *n* = 0; abstain, *n* = 0. Agreement = 100%

A prospective randomized cadaveric study has shown that US-guided injections in the pes anserinus bursa are feasible and more accurate than palpation-guided injections [79]. A prospective controlled study comparing US-guided to blind corticosteroid injections into the pes anserinus bursa of patients with bursitis showed that the US-guided injections resulted in greater improvement at 1 and 4 weeks compared to blind injections [80]. Based on review papers, possible interventions in the prepatellar bursa include US-guided corticosteroid injection into an inflamed bursa or aspiration for diagnosis of infection or other synovial pathology [81]. However, no information about efficacy is available. Smith et al. showed 83–100% accuracy of needle placement into the popliteus tendon sheath. Finnoff et al. achieved 92% accuracy in injecting the pes anserinus bursa with US guidance but only 17% accuracy using landmark injections [79]. Jose et al. injected corticosteroid and anesthetic into the medial collateral ligament bursa [82], while Hong et al. used the same mixture for iliotibial band syndrome [83]. More studies comparing guidance modalities and corticosteroids to other therapies are needed.

## Discussion

We found some evidence concerning image-guided procedures around the knee. In all statements, US guidance has been established as pivotal, as accuracy and clinical outcome are generally higher compared to palpation-guided procedures with the highest level of evidence (statement #1). Moreover, US-guided injection of corticosteroid-anesthetic has proven to be effective in the short-to-midterm follow-up for treating inflammatory arthritis with a level of evidence 2 (statement #2). Conversely, despite being seemingly safe and effective in treating osteoarthritis, strong evidence is still lacking for US-guided injections of HA (statement #3) and regenerative medications (statement #4). Furthermore, only small case series are available for US-guided procedures around the menisci and injections and alcohol ablation in knee anterior fat pad–related syndromes, reporting promising results that require to be further confirmed by larger series.

Regarding periarticular treatments, PT is the most investigated topic. According to our results, US-guided dry needling is highly effective in PT with a higher level of evidence (statement #7). Notably, although the association of PRP to dry needling seems to improve the outcome, there are still conflicting results concerning the value of PRP alone for PT, leading us to recommend avoiding this treatment as a first-line strategy. Although different safe and effective US-guided treatment options exist, no randomized prospective studies have effectively clarified which one should be preferred as the best choice (statement #8). Last, US-guided treatments of Baker’s cyst (statement #9) and pes anserinus bursitis (statement #10) are both safe and effective, but the level of evidence of these procedures is still too low to strongly recommend these treatments.

In summary, ten statements regarding US-guided musculoskeletal interventions around the knee have been provided by a working group of experts from the ESSR. US guidance is strongly recommended for intra-articular and PT procedures to ensure higher accuracy and efficacy. Prospective randomized studies remain warranted, especially for knee procedures with low levels of evidence.

## Supplementary Information

Below is the link to the electronic supplementary material.

Supplementary file1 (DOCX 21 KB)

## Abbreviations

ESSR: European Society of Musculoskeletal Radiology
HA: Hyaluronic acid
PRP: Platelet-rich plasma
PT: Patellar tendinopathy
US: Ultrasonography

## Declarations

### Guarantor

The scientific guarantor of this publication is Luca Maria Sconfienza, MD PhD.

### Statistics and Biometry

No complex statistical methods were necessary for this paper.

### Methodology

- Literature-based Delphi process
